# Comprehensive study of 28 individuals with *SIN3A*-related disorder underscoring the associated mild cognitive and distinctive facial phenotype

**DOI:** 10.1038/s41431-020-00769-7

**Published:** 2021-01-12

**Authors:** Meena Balasubramanian, Alexander J. M. Dingemans, Shadi Albaba, Ruth Richardson, Thabo M. Yates, Helen Cox, Sofia Douzgou, Ruth Armstrong, Francis H. Sansbury, Katherine B. Burke, Andrew E. Fry, Nicola Ragge, Saba Sharif, Alison Foster, Annachiara De Sandre-Giovannoli, Sahar Elouej, Pradeep Vasudevan, Sahar Mansour, Kate Wilson, Helen Stewart, Solveig Heide, Caroline Nava, Boris Keren, Serwet Demirdas, Alice S. Brooks, Marie Vincent, Bertrand Isidor, Sebastien Küry, Meyke Schouten, Erika Leenders, Wendy K. Chung, Arie van Haeringen, Thomas Scheffner, Francois-Guillaume Debray, Susan M. White, Maria Irene Valenzuela Palafoll, Rolph Pfundt, Ruth Newbury-Ecob, Tjitske Kleefstra

**Affiliations:** 1 Sheffield Clinical Genetics Service, Sheffield Children’s NHS Foundation Trust, Sheffield, UK; 2 Academic Unit of Child Health, Department of Oncology & Metabolism, University of Sheffield, Sheffield, UK; 3 Department of Human Genetics, Donders Institute for Brain, Cognition and Behavior, Radboud University Medical Center, Nijmegen, the Netherlands; 4 Sheffield Diagnostic Genetics Service, Sheffield Children’s NHS Foundation Trust, Sheffield, UK; 5 Northern Genetics Service, Newcastle upon Tyne Hospitals NHS Trust, Newcastle, UK; 6 West Midlands Regional Clinical Genetics Service and Birmingham Health Partners, Birmingham Women’s and Children’s Hospitals NHS Foundation Trust, Birmingham, UK; 7 Manchester Centre for Genomic Medicine, Saint Mary’s Hospital, Manchester University NHS Foundation Trust, Manchester, UK; 8 Division of Evolution and Genomic Sciences, School of Biological Sciences, Faculty of Biology, Medicines and Health, University of Manchester, Manchester, UK; 9 East Anglian Medical Genetics Service, Addenbrooke’s Hospital, Cambridge, UK; 10 All Wales Medical Genomics Service, NHS Wales Cardiff and Vale University Health Board, Institute of Medical Genetics, University Hospital of Wales, Cardiff, UK; 11 Department of Biological and Medical Sciences, Oxford Brookes University, Oxford, UK; 12 Aix Marseille Univ, INSERM, MMG, U1251 Marseille, France; 13 Department of Medical Genetics, La Timone Children’s Hospital, Marseille, France; 14 Biological Resource Center (CRB-TAC), Assistance Publique Hôpitaux de Marseille, La Timone Children’s Hospital, Marseille, France; 15 Leicester Clinical Genetics Service, University Hospitals of Leicester NHS Trust, Leicester, UK; 16 Clinical Genetics Service, St George’s University Hospitals NHS Foundation Trust, London, UK; 17 Oxford Centre for Genomic Medicine, Nuffield Orthopaedic Centre, Oxford University Hospitals NHS Foundation Trust, Oxford, UK; 18 Clinical Genetics Service, GH Pitié-Salpêtrière, Pitié Salpêtrière Hospital, APHP Sorbonne University, Paris, France; 19 Department of Clinical Genetics, Erasmus Medical Centre, Erasmus University, Rotterdam, the Netherlands; 20 Service de Génétique Médicale, CHU de Nantes, 44000 Nantes, France; 21 Inserm, CNRS, Univ Nantes, l’institut du thorax, 44000 Nantes, France; 22 Departments of Pediatrics and Medicine, Columbia University, New York, USA; 23 Department of Clinical Genetics, Leiden University Medical Center, Leiden, the Netherlands; 24 Klinik für Kinder- und Jugendmedizin, Perinatal- und Stoffwechselzentrum, Reutlingen, Germany; 25 Metabolic Unit—Department of Medical Genetics, CHU & University Liège Domaine L Sart-Tilman Bât B35, B-4000 Liège, Belgium; 26 Victorian Clinical Genetics Services, Murdoch Children’s Research Institute, Melbourne, VIC Australia; 27 Department of Paediatrics, University of Melbourne, Melbourne, VIC Australia; 28 Department of Clinical and Molecular Genetics, University Hospital Vall d´Hebron and Medicine Genetics Group, Valle Hebron Research Institute, Barcelona, Spain; 29 University Hospitals Bristol NHS Foundation Trust, Clinical Genetics, St. Michael’s Hospital, Bristol, UK

**Keywords:** Autism spectrum disorders, Genetic testing

## Abstract

Witteveen-Kolk syndrome (OMIM 613406) is a recently defined neurodevelopmental syndrome caused by heterozygous loss-of-function variants in *SIN3A*. We define the clinical and neurodevelopmental phenotypes related to *SIN3A*-haploinsufficiency in 28 unreported patients. Patients with *SIN3A* variants adversely affecting protein function have mild intellectual disability, growth and feeding difficulties. Involvement of a multidisciplinary team including a geneticist, paediatrician and neurologist should be considered in managing these patients. Patients described here were identified through a combination of clinical evaluation and gene matching strategies (GeneMatcher and Decipher). All patients consented to participate in this study. Mean age of this cohort was 8.2 years (17 males, 11 females). Out of 16 patients ≥ 8 years old assessed, eight (50%) had mild intellectual disability (ID), four had moderate ID (22%), and one had severe ID (6%). Four (25%) did not have any cognitive impairment. Other neurological symptoms such as seizures (4/28) and hypotonia (12/28) were common. Behaviour problems were reported in a minority. In patients ≥2 years, three were diagnosed with Autism Spectrum Disorder (ASD) and four with Attention Deficit Hyperactivity Disorder (ADHD). We report 27 novel variants and one previously reported variant. 24 were truncating variants; three were missense variants and one large in-frame gain including exons 10–12.

## Introduction

Witteveen-Kolk syndrome (OMIM #613406) was first described in 2016 with characteristic distinctive facial features, microcephaly, short stature, mild intellectual disability (ID) with delayed cognitive and motor development and subtle anomalies on MRI-brain imaging [1]. Although sparsely reported, frameshift as well as missense variants in *the Switch-insensitive 3 transcription regulator family member A (SIN3A)* (OMIM *607776) have been described in larger neurodevelopmental disorder cohorts, with an overall mild clinical picture [2]. Narumi-Kishimoto et al. presented a further patient with *SIN3A* frameshift variant and facial features of Witteveen-Kolk with relatively mild ID and normal growth [3].

The *SIN3A* gene is located in the chromosome 15 band q24 and is within the shortest region of overlap of various reported 15q24 microdeletions, therefore, is thought to be the critical gene for the atypical 15q24 microdeletion syndrome [4]. *SIN3A* encodes a transcriptional regulatory protein, which is associated with scaffolding in the core histone deacetylase complex [5]. In our earlier study, we showed that SIN3A is involved in cortical neurogenesis, supporting the hypothesis that variants in the gene that adversely affect its function lead to a broad range of developmental and neurological problems. We identified an additional 28 patients with *SIN3A* variants in order to comprehensively define the phenotype with a focus on both developmental and behavioural aspects, as well as investigating genotype-phenotype correlations.

## Methods

We collected the molecular and clinical features on 28 unpublished individuals with *SIN3A* variants by a collaboration facilitated by Deciphering Developmental Disorders (DDD study) [6], GeneMatcher [7] and DECIPHER (DECIPHER v9.24: https://decipher.sanger.ac.uk/) [8], in which, several clinical groups independently identified individuals with developmental delay/intellectual disability (DD/ID) and related phenotypes with rare variants in *SIN3A* during routine diagnostic genetic testing. An application to the DDD study for a Complementary Analysis Project was made, allowing access to anonymised details of patients with *SIN3A* variants identified through this study (https://www.ddduk.org/). Clinicians of selected patients were then contacted to invite patients and their families to be recruited. Clinical analysis of these patients was performed during regular consultations focusing on medical history, physical examination and observational analysis of behavioural features along with reported history by the family. In all patients, exome sequencing and variant filtering were performed, according to the routine protocol and diagnostic procedures at each institute.

Identified patients with a class 4 or 5 variants (likely pathogenic or pathogenic variants) in *SIN3A*, according to the American College of Medical Genetics (ACMG) criteria, were approached to participate in this study. Informed consent for publication was obtained from all patients and/or their guardians. The responsible clinician reviewed medical records of each participant in order to comprehensively document the phenotype.

The clinical significance of the variants identified was interpreted according to the ACMG [9] guidelines and further review publications [10]. Excluded from the study were: patients with an additional proven genetic diagnosis where the *SIN3A* variant was not thought to be contributory or the sole pathogenic finding, those with a chromosomal anomaly explaining or likely to be explaining the phenotype, and those in whom *SIN3A* variants were of uncertain clinical significance with no convincing clinical correlation. All the variants reported here have been submitted to DECIPHER database [https://decipher.sanger.ac.uk] and phenotypes are included in DECIPHER database for the DDD patients [Patients 1–13]- DDD identifiers, [https://decipher.sanger.ac.uk/gene/SIN3A/overview/clinical-info].

## Results

### Molecular genetics

**Fig. 1:**
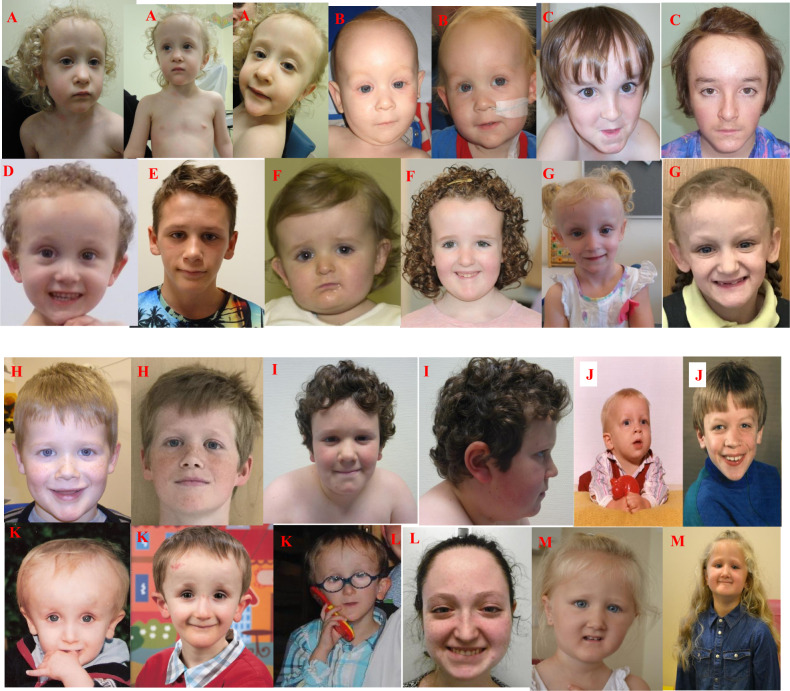
Characteristic facial appearance of patients with variants in SIN3A. Note the high forehead, small, pointed chin and down-slanting palpebral fissures. **A**: Patient 1, **B** Patient 2, **C** Patient 4, **D** Patient 5, **E** Patient 9, **F** Patient 11, **G** Patient 12, **H** Patient 13, **I** Patient 16, **J** Patient 18, **K** Patient 25, **L** Patient 27, **M** Patient 28.

In this study, we reported 28 patients with variants that adversely affect protein function and which are classified as pathogenic or likely pathogenic variants in *SIN3A*. The findings of this study expand the variant spectrum previously reported in *SIN3A*^,3^. 27/28 patients in this study have novel variants. Patient 15 with c.3310C>T, p.(Arg1104*) variant is a family member of a patient we have previously reported in Witteveen et al. [1]. Predominantly, variants found in our patient cohort are truncating and predicted to result in a protein loss-of-function (25/28). All 24 truncating variants have been classified as variants that adversely affect function (pathogenic) using the ACMG and ACGS (The Association for Clinical Genomics Science) variant classification guidelines using criterions: PVS1, PM2, and PS2 where appropriate [9, 10]. Of the four remaining variants, three were missense (patients 4, 18 and 26; Table 1) and one was a large in-frame gain which included the whole of exons 10, 11 and 12 (patient 24; Table 1). These four variants were all de novo in our patients with a specific and consistent phenotype to *SIN3A* and were absent from control population data sets in gnomAD (https://gnomad.broadinstitute.org/). We have also used criterion PM1 at moderate level for two of the three reported missense variants, c.377C>T, p.(Ala126Val) and c.463A>G, p.(Lys155Glu), as they were located within a protein functional domain. These results show that missense causative variants are not clustered in a hot spot within *SIN3A* (Fig. 1 provides a schematic SIN3A structure and variant locations which, as demonstrated, are distributed throughout the gene). Details of the variants, ACMG criterions and classification are listed in Table 1.

**Table 1:** Variant Table.

No	Variant using NM_001145358.1	Inheritance	ACMG criterion	Classification	Published	DECIPHER ID
1	c.1462del; p.(Val488Leufs*7)	De novo	PS2-T/PVS1/PM2-M	Pathogenic	Novel	278680
2	c.2764C>T; p.(Arg922*)	De novo	PS2-T/PVS1/PM2-M	Pathogenic	Novel	306260
3	c.588del; p.(Asn197Metfs*4)	De novo	PS2-T/PVS1/PM2-M	Pathogenic	Novel	271952
4	c.377C>T; p.(Ala126Val)	De novo	PS2-T/PM1-M/PM2-M/PP3-S	Likely Pathogenic	Novel	262798
5	c.1245_1246del; p.(Asn415Lysfs*24)	De novo	PS2-T/PVS1/PM2-M	Pathogenic	Novel	293976
6	c.775dup; p.(His259Profs*47)	De novo	PS2-T/PVS1/PM2-M	Pathogenic	Novel	263709
7	c.824del; p.(Pro275Hisfs*12)	De novo	PS2-T/PVS1/PM2-M	Pathogenic	Novel	307508
8	c.2248_2251del; p.(Leu750Metfs*43)	De novo	PS2-T/PVS1/PM2-M	Pathogenic	Novel	266515
9	c.2339del; p.(Ala780Glyfs*14)	De novo	PS2-T/PVS1/PM2-M	Pathogenic	Novel	260388
10	c.889C>T; p.(Gln297*)	De novo	PS2-T/PVS1/PM2-M	Pathogenic	Novel	264582
11	c.1715_1722delinsCCCAAGTGTA; p.(Gly572Alafs*11)	De novo	PS2-T/PVS1/PM2-M	Pathogenic	Novel	292229
12	c.3323C>G; p.(Ser1108*)	De novo	PS2-T/PVS1/PM2-M	Pathogenic	Novel	282105
13	c.3490A>T; p.(Lys1164*)	De novo	PS2-T/PVS1/PM2-M	Pathogenic	Novel	282212
14	c.46C>T; p.(Gln16*)	Maternal	PVS1/PM2-M	Pathogenic	Novel	421224
15	c.3310C>T; p.(Arg1104*)	Unknown	PVS1/PM2-M	Pathogenic	Witteveen et al. [1]	421225
16	c.2809_2810del; p.(Lys937Glufs*2)	De novo	PS2-T/PVS1/PM2-M	Pathogenic	Novel	421226
17	c.1489_1499del; p.(Arg497Cysfs*13)	De novo	PS2-T/PVS1/PM2-M	Pathogenic	Novel	421228
18	c.3317T>C; p.(Met1106Thr)	De novo	PS2-T/PM2-M/BP4-S	Likely Pathogenic	Novel	421229
19	c.1675C>T; p.(Arg559*)	De novo	PS2-T/PVS1/PM2-M	Pathogenic	Novel	421230
20	c.3303C>G; p.(Tyr1101*)	De novo	PS2-T/PVS1/PM2-M	Pathogenic	Novel	421231
21	c.1570_1577del; p.(Tyr524Valfs*26)	De novo	PS2-T/PVS1/PM2-M	Pathogenic	Novel	421232
22	c.2185_2186del; p.(Leu729Glyfs*8)	De novo	PS2-T/PVS1/PM2-M	Pathogenic	Novel	421233
23	c.3441_3445del; p.(Lys1148Argfs*12)	De novo	PS-2-T/PVS1/PM2-M	Pathogenic	Novel	421234
24	c.1318_1737dup; p.(Val580Lysfs*35)	De novo	PS2-T/PVS1	Pathogenic	Novel	421236
25	c.1773G>A; p.(Trp591*)	De novo	PS2-T/PVS1/PM2-M	Pathogenic	Novel	421237
26	c.463A>G; p.(Lys155Glu)	De novo	PS2-T/PM1-M/PM2-M/PP3-S	Likely Pathogenic	Novel	421238
27	c.2803C>T; p.(Arg935*)	De novo	PS-2T/PVS1/PM2-M	Pathogenic	Novel	421239
28	c.1888dup; p.(Ile630Asnfs*17)	De novo	PS-2/PVS1/PM2-M	Pathogenic	Novel	421240

ACMG Criterion applied:

PS2-T: De novo (both maternity and paternity confirmed) in a patient with the disease and no family history, used at strong level.

PVS1: null variant (nonsense, frameshift, canonical ±1 or 2 splice sites, initiation codon, single or multiexon deletion) in a gene where LOF is a known mechanism of disease.

PM1-M: Located in a critical functional domain without benign variation, used at moderate level.

PM2-M: Absent from controls in gnomAD database, used at moderate level.

PP3-S: Multiple lines of computational evidence support a deleterious effect on the gene or gene product, used at supporting level.

PM4-M: Protein length changes as a result of in-frame deletions/insertions in a non-repeat region or stop-loss variants, used at moderate level.

BP4-S: Multiple lines of computational evidence suggest no impact on gene or gene product, used at supporting level

No corresponds to patient number.

DECIPHER ID corresponds to entry of open access variant on https://decipher.sanger.ac.uk [DatabasE of genomiC varIation and Phenotype in Humans using Ensembl Resources].

### Assessment of pathogenicity of missense variants in *SIN3A*

In order to assess if missense variants in our cohort were predicted to adversely affect the critical functional domains in SIN3A, we used a 3D model based on the solution structure of mouse SIN3A PAH1 bound to the Sin3 interaction domain (SID) of SAP25 (SIN3A Associated Protein 25) [11]. The pair of amphipathic helices (PAH) domains are predicted as important for the recruitment by and interaction with a diverse number of transcription factors and therefore, critical for protein function [12, 13]. Both altered residues by c.377C>T, p.(Ala126Val) and c.463A>G, p.(Lys155Glu) were predicted to be part of the SAP25 SID-binding surface. We predicted that SIN3A Ala126 with its small hydrophobic side chain formed a pocket for the larger hydrophobic side chain of SAP25 Leu142 and was invariant across species [11].

**Fig. 2:**
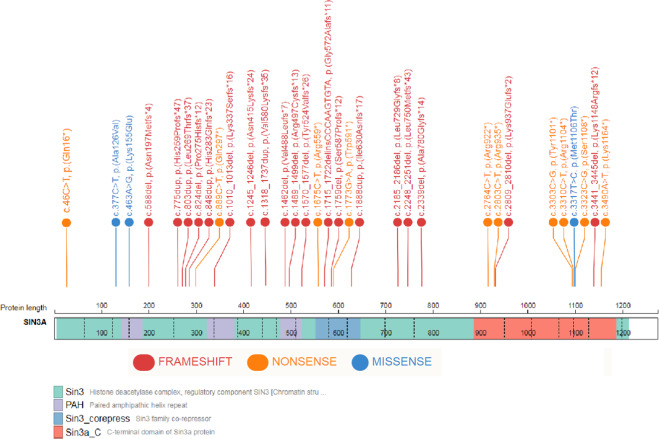
Schematic SIN3A protein structure and variant location. ITD: intragenic deletion; Plot of variants done using St. Jude Cloud protein paint (https://pecan.stjude.cloud/proteinpaint).

SIN3A Lys155 is also predicted to form a 2.2A hydrogen bond with the polar Gln143 of SAP25 (orange line in Fig. 2) and was also conserved in the PAH1 domain across species. Along with the lack of normal variation in this region (using missense constraint data in SIN3A from Decipher: https://decipher.sanger.ac.uk/gene/SIN3A#overview/protein-info), we applied criterion PM1 at moderate level for the classification of both c.377C>T, p.(Ala126Val) and c.463A>G, p.(Lys155Glu). We also suggest that PM1 can be applied at a moderate level for missense variants within residues p.119–189 of the SIN3A protein providing that the change caused by the variant was not present in other species and also meets the PM2 criteria (see Fig. 2).

Finally, the c.3317T>C, p.(Met1106Thr) is within the C-terminal domain of SIN3A, this region is not well characterised and the in silico pathogenicity prediction programs suggests a benign effect (REVEL score of 0.105 shows a benign effect). However, this variant was confirmed to be de novo in patient 18, novel in control populations in gnomAD and with a consistent phenotype to that of the rest of our cohort. No other variants were found in this patient on exome sequencing. Therefore, we classified this variant in addition as likely pathogenic, but without the use of PM1.

### Clinical findings

Table 2 shows a summary of the characteristics of 28 patients (17 males, 11 females) with variants in *SIN3A* who were included in this retrospective study. The mean age of participants was 8.2 years (range 0.6–67 years). Table 3 provides an overview of salient features described in this cohort in comparison to published literature and Table 4 has detailed phenotypic information on this cohort of patients.

**Table 2:** Select phenotypic characteristics of SIN3A cohort compared with previously published literature.

Patient	Sex/age	Variant	Development	Psychopathology	Neurological features	Feeding Difficulties	Growth abnormality	Other somatic features
1 DECIPHER 278680	F/9 y	c.1462del, p.(Val488Leufs*7)	GDD; ataxia; delayed speech	N/A	Hypotonia Cerebellar atrophy	Yes	Weight & OFC −2SD Height −1SD	Scoliosis; strabismus
2 DECIPHER 306260	M/1.2 y	c.2764C>T, p.(Arg922*)	Language-and motor delay	?ASD	Hypotonia?; epilepsy Foci high signal white matter, dilation of lateral ventricles	Yes	Weight < −2SD OFC < −2SD	Apnoea; Cow’s milk protein intolerance, nystagmus secondary to oculocutaneous albinism
3 DECIPHER 271952	F/8.3 y	c.588del, p.(Asn197Metfs*4)	Mild ID, language-and motor delay	ADHD	Hypotonia	Yes	No	
4 DECIPHER 262798	M/13.5 y	c.377C>T, p.(Ala126Val)	GDD language-and motor delay, Developmental coordination disorder	Aggressive behaviour, High functioning autism	Neonatal hypotonia	Yes	No	Constipation; sacral dimple, Hirsute on back as baby; pes planus joint hypermobility hearing impairment (mixed)
5 DECIPHER 293976	M/10.1 y	c.1245_1246del, p.(Asn415Lysfs*24)	Moderate ID, language and motor delay	None	Neonatal hypotonia	Yes	OFC < −2SD	Pectus excavatum. Unilateral inguinal hernia; Sacral dimple
6 DECIPHER 263709	M/5.4 y	c.775dup, p.(His259Profs*47)	GDD, language delay	None	None	Unknown	Height < −2SD Weight < −2SD OFC < -2SD	Multiple dental caries; Eczema Failure to thrive; Mild conductive hearing impairment, Obstructive sleep apnoea, Submucous cleft hard palate
7 DECIPHER 307508	M/0.6 y	c.824del, p.(Pro275Hisfs*12)	GDD, language delay	N/A	Hypotonia	No	Height < −2SD Weight < −2SD OFC < −2SD	Sensorineural hearing loss
8 DECIPHER 266515	M/7.2 y	c.2248_2251del, p.(Leu750Metfs*43)	GDD, language-and motor delay	None	Hypotonia	Yes	Weight < −2SD OFC < −2SD	
9 DECIPHER 260388	M/5 y	c.2339del, p.(Ala780Glyfs*14)	GDD language and motor delay	Hyperactive behaviour	None	Yes	Height < −2SD Weight < −2SD OFC < −2SD	Poor sleep; Oromotor coordination dysfunction
10 DECIPHER 264582	M/3.2 y	c.889C>T, p.(Gln297*)	GDD, language-and motor delay	None	None	No	Height unknown Weight unknown OFC < −2SD	Aplasia cutis congenita over the scalp vertex; Capillary hemangiomas; Reduced subcutaneous adipose tissue
11 DECIPHER 292229	F/7.9	c.1715_1722delinsCCCAAGTGTA, p.(Gly572Alafs*11)	GDD, language delay	Immature behaviour;?ASD	Febrile seizures	No	No	
12 DECIPHER 282105	F/4.6	c.3323C>G, p.(Ser1108*)		ADHD, Aggressive behaviour, recurring obsessions		Yes	Height < −2SD Weight < −2SD OFC < −2SD	
13 DECIPHER 282212	M/9.1	c.3490A>T, p.(Lys1164*)	Mild ID, language delay	ADHD		No	OFC < −2SD	
14 DECIPHER 421224	M/25 y	c.46C>T, p.(Gln16*)	Moderate ID, GDD, language-and motor delay	None	Epilepsy, thinning of the corpus callosum	No	No	
15 DECIPHER 421225	F/67 y	c.3310C>T,p.(Arg1104*)	Not formally assessed, possible mild ID	None	None	Unknown	OFC < −2SD	2 episodes DVT cystocele and urethrocele, EVAR prosthesis, hernia cicatricalis, sigmoïd adenocarcinoma
16 DECIPHER 421226	M/11.1 y	c.2809_2810del,p.(Lys937Glufs*2)	Mild ID, GDD, language- motor delay	None	None	No	OFC < -2SD	Scoliosis; Burkitt lymphoma (at 8 y)
17 DECIPHER 421228	F/8.1 y	c.1489_1499del, p.(Arg497Cysfs*13)	Mild ID,GDD, language- motor delay	None	Sleeping problems	Yes	Height < −2SD Weight < −2SD OFC < −2SD	Orofacial cleft; conductive hearing loss
18 DECIPHER 421229	M/36.2 y	c.3317T>C,p.(Met1106Thr)	Moderate ID, language-motor delay	None	Epilepsy	No	No	
19 DECIPHER 421230	M/22.1 y	c.1675C>T,p.(Arg559*)	Mild ID	ASD, depression, psychosis	Sleeping problems	Yes	No	Thickened aortic valve
20 DECIPHER 421231	F/24.2 y	c.3303C>G,p.(Tyr1101*)	Language delay	Multiple Complex DD, anxiety disorder	Hypotonia	Yes	No	
21 DECIPHER 421232	F/19 y	c.1570_1577del, p.(Tyr524Valfs*26)	Normal	Schizoaffective disorder	Neonatal hypotonia	No	Unknown	Pelvic kidney; Palate defect; Congenital dislocation of the hip
22 DECIPHER 421233	M/10 y	c.2185_2186del, p.(Leu729Glyfs*8)	Mild motor delay	None	Hypotonia, epilepsy	Unknown	No	
23 DECIPHER 421234	F/0.6 y	c.3441_3445del,p.(Lys1148Argfs*12)	Mild motor delay	N/A	Hypotonia	Yes	No	
24 DECIPHER 421236	M/1.5 y	c.1318_1737dup; p.(Val580Lysfs*35)	GDD	N/A	Hypotonia, epilepsy	Yes	Unknown	
25 DECIPHER 421237	M/6 y	c.1773G>A, p.(Trp591*)	Language and motor delay	None	Enlarged cerebral spaces	Yes	Weight < −2SD	
26 DECIPHER 421238	M/11.3 y	c.463A>G, p.(Lys155Glu)	Severe ID, non-verbal	ASD	Hypotonia Dysplastic corpus callosum Ventriculomegaly	Yes	No	Multiple VSDs; bilateral iris and chorioretinal coloboma hearing loss; (mixed) immunodeficiency
27 DECIPHER 421239	F/19 y	c.2803C>T, p.(Arg935*)	Mild ID, mild language and motor delay	ADHD	Hypotonia Headaches Chiari I malformation	No	No	Joint laxity, recurrent otitis media; asthma; scoliosis
28 DECIPHER 421240	F/6.5 y	c.1888dup, p.(Ile630Asnfs*17)	Mild GDD,?mild ID	None	Hypotonia	No	OFC < −2SD	Joint laxity; Unilateral hypermetropia; multiple dental caries
Witteveen et al. [1]								
1. Pt 5	M/9.1 y	c.803dup, p.(Leu269Thrfs*37)	Moderate - severe ID, language- and motor delay	ASD	Hypotonia, epilepsy, central apnoea syndrome			
2. Pt 6	F/16.4 y	c.1010_1013del, p.(Lys337Serfs*16)	Mild ID, motor delay	ASD, depression, PTSS	Hypotonia, epilepsy			
3. Pt 7	M/16 y	c.1759del, p.(Ser587Profs*12)	Mild-moderate dev. delay	None reported	None			
4. Pt 8	F/3 m	c.1759del, p.(Ser587Profs*12)	Moderate ID, language-and motor delay	None reported	None			
5. Pt 9	Father	c.1759del, p.(Ser587Profs*12)	No dev. delay reported	None reported	None reported			
6. Pt 10	M/9.6 y	c.1759del, p.(Ser587Profs*12)	Mild-moderate dev. delay	ID, ASD	None reported			
7. Pt 11	M/4 y	c.3310C>T,p.(Arg1104*)	Mild-moderate dev. delay	ID	None reported			
8. Pt 12	M/9 m	c.3310C>T,p.(Arg1104*)	Mild-moderate dev. delay	ID	None reported			
9. Pt 13	Mother	c.3310C>T,p.(Arg1104*)	No dev. delay reported	?ID	None reported			
10. Narumi-Kishimoto Pt	F/7 y	c.848dup, p.(His283Glnfs*23)	Moderate dev. delay	ID, ASD, poor moto co-ordination	None reported			

*ID* intellectual disability, *ASD* autism spectrum disorder, *ADHD* attention deficit hyperactivity disorder, *GDD* global developmental delay.

**Table 3:** Summary of salient features in SIN3A-related disorder based on current cohort and previously published literature.

Clinical feature	Current cohort (*n* = 28)	Witteveen et al. [1] (*n* = 9)	Narumi-Kishimoto [3] (*n* = 1)	Total (*n* = 38)
Intellectual disability	16	7	1	24
Mild	8	5	–	13
Moderate	7	2	1	10
Severe	1	–	–	1
Speech delay	11	2	1	13
Hypotonia	12	2	–	14
Feeding difficulties	15	N/R	–	15
Short stature	6	2	–	8
Epilepsy	4	1	–	5
Behavioural problems	12	4	1	17

*N/R* not reported, overlap between mild-moderate *ID* reported as mild.

### Development

Of 28 patients, 15 (56%) had global developmental delay. Out of 16 patients ≥8 years old, (one patient was not formally assessed) seven (44%) had mild intellectual disability (ID), four had moderate ID (25%), and one had severe ID (6%). Four (25%) did not appear to have any cognitive impairment. In 21 patients ≥5 years old, some form of motor developmental delay was reported in 13 patients (62%) and in 16 patients (76%) there was some form of language developmental delay. Intelligence Quotient (IQ) was only formally measured in 6/28 patients and the score ranged from 60 to 100. This suggests that patients within this cohort have low normal intelligence.

Autism spectrum disorder (ASD) is rarely diagnosed before the age of 24 months [14]. In one patient aged 14 months, ASD was thought possible but not formally assessed due to the young age. In three of 23 patients ≥2 years (13%), ASD was diagnosed. Attention Deficit Hyperactivity Disorder (ADHD) is most commonly diagnosed in children between 6 and 12 years, though this can be diagnosed in a younger age group. ADHD was diagnosed in four patients ≥2 years (17%).

### Growth and feeding difficulties

13/28 (46%) patients in our cohort had head circumference at least two standard deviations (SD) below the mean. Weight was less than two SD below the mean in 8/28 patients (29%) and height was less than two SD below the mean in 5/28 patients (18%).15/28 (54%) of patients had feeding difficulties with at least two patients documented to require nasogastric tube feeding mainly in the neonatal period.

### Craniofacial features

**Fig. 3:**
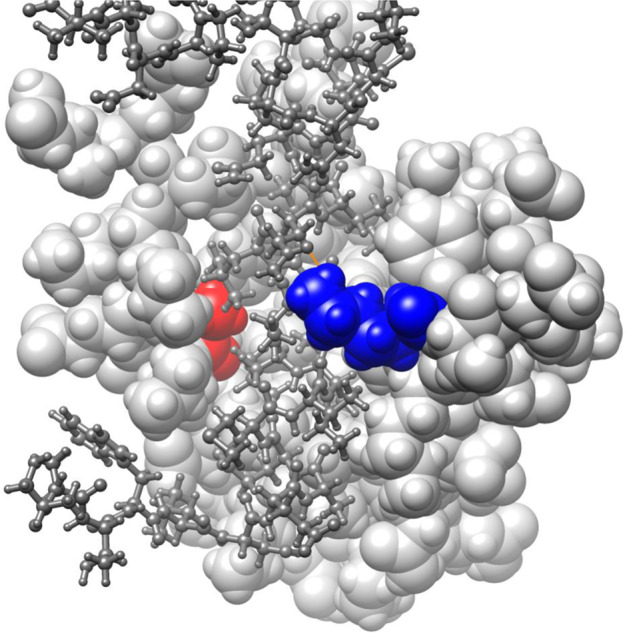
3D model to demonstrate predicted consequences of SIN3A missense variant in our cohort. This 3D-model is based on the solution structure of mouse Sin3A PAH1 bound to the Sin3 interaction domain (SID) of SAP25 (Sin3A Associated Protein 25) displayed using UCSF Chimera v1.14 (Pattersen et al. 2004). The PAH1 domain of Sin3A (residues 119-189; sphere model, light grey) with residues Ala126 (red) and Lys155 (blue) highlighted. The SAP25 protein SID domain (residues 126-186; ball and stick model, dark grey) binds in the fold formed by the four helices of Sin3A PAH1. Sin3A Lys155 is predicted to form a 2.2A hydrogen bond with the polar side chain of Gln143 of SAP25 (orange line).

Fourteen (50%) patients were reported to have craniofacial dysmorphism. In ten patients, the facial gestalt was confirmed independently by two clinical geneticists with expertise in dysmorphology. Common facial features included a broad, tall forehead; small mouth, thin upper lip with pointed chin and down-slanting palpebral fissures (see Fig. 3). The facial gestalt appears to be similar and potentially recognisable but only in the context of reverse phenotyping with published images, confirming previous findings. Three patients had a palatal defect with one also having a bifid uvula.

### Other clinical manifestations

In terms of neuroimaging, given the milder developmental delay phenotype, many patients in the cohort have not had MRI-brain imaging (19/28, 68%). Nine out of a total of nine patients that had brain imaging performed had reports available and in 7/9 (78%) abnormalities were seen. Most common was ventriculomegaly in 2/9, and a hypoplastic/dysplastic corpus callosum in 2/9. Cerebellar atrophy was reported in two patients and there was one patient had a Chiari 1 malformation.

6/28 (21%) patients were noted to have seizures currently or in their past medical history. Hypotonia was a common manifestation as well, present in 12/28 (43%) patients.

Other reported features included hearing loss (5/28; 18%) of which 1 patient had sensorineural hearing loss, two patients had conductive hearing loss and two patients had mixed hearing loss. Ocular abnormalities reported included strabismus (1), nystagmus secondary to ocular albinism (1), bilateral iris and chorioretinal coloboma (1) and hypermetropia (1).

Of note, 2/28 patients were reported to have a malignancy, including sigmoid adenocarcinoma in the 67-year old patient which may be an age-related cancer and 1 younger patient with Burkitt lymphoma (Patient 16 at age eight). Patient 26 was noted to have T-cell lymphopenia at age 11 associated with immunoglobulin deficiency and significant bronchiectasis.

## Discussion

Heterozygous loss-of-function variants in *SIN3A* were recently described to result in a novel neurodevelopmental syndrome comprising intellectual disability and varying degrees of developmental delay. This syndrome defined as Witteveen-Kolk syndrome was further characterised by subtle brain abnormalities, including corpus callosum dysgenesis and ventriculomegaly, distinctive facial features (a broad, tall forehead; small mouth, thin upper lip with pointed chin and down-slanting palpebral fissures), hyperlaxity and short stature. Furthermore, we showed previously that in vivo functional knockdown of *SIN3A* leads to reduced cortical neurogenesis, altered neuronal identity and aberrant cortico-cortical projections in the developing mouse brain. Therefore, it is likely that the aberrant cortical development underlies impaired neurodevelopment and leads to cognitive and behavioural problems in patients, varying from paediatric to adult-onset. Here, we summarise the neurodevelopmental and facial phenotype in an additional 28 patients with *SIN3A*-related disorder.

Table 2 provides an overview of the patients reported here and all previously reported patients with *SIN3A* structural variants alone. Supplementary table provides a comprehensive review of all the clinical information available on this cohort. Below is extracted information from these tables.

### Genotype-phenotype correlation in *SIN3A*

Most of the patients in this cohort (25/28; 89%) have truncating or frameshift variants in *SIN3A* except three patients with missense variants (Patients 4, 18 and 26) which have been classed as a class 4 (likely pathogenic) variant based on a combination of evidence as previously described (see variant table for further information). It is likely that as previously described haploinsufficiency is the predominant likely mechanism in SIN3A-related disorder but it may be that the phenotype may differ depending on the nature of the *SIN3A* variant. From the evidence gathered so far, there is no apparent correlation between severity of phenotype and genotype.

### Neurodevelopment

As noted in our earlier study [1], the overall intellectual disability seems to be mild, with 11 of the 16 patients (69%) over the age of eight years having either no ID or mild ID. The low prevalence of severe intellectual disability different from the neurodevelopmental phenotype of moderate or severe intellectual disability associated with 15q24 microdeletion syndrome, suggests that additional genes contribute to the cognitive phenotype in the 15q24 deletion syndrome [15].

The median intelligence is relatively high with IQ of 74. Interestingly, all patients in whom intelligence was tested reported a higher verbal than performal intelligence score. This is an important finding that clinicians should be aware of, since disharmonic intelligence profiles easily lead to overestimating of the self-management capabilities of patients. A further study is planned to undertake detailed psychometric assessments and IQ measurements in this cohort.

### Behavioural phenotype

Overall, a third of the cohort had a psychiatric or behavioural condition reported, including ADHD, aggressive behaviour, OCD, depression, psychosis, anxiety and schizoaffective disorder. In three patients, ASD was concurrent with a psychiatric diagnosis. This is a significant finding, since such neuropathology has a significant impact on the quality of life of patients especially in those adults with milder neurodevelopmental phenotypes. Knowing that patients with variants in the *SIN3A* gene are at risk for such concerns, early intervention is important to ensure optimal treatment and outcomes.

Of those patients with psychiatric disease, only two had brain imaging done. Interestingly, both those MRIs showed ventriculomegaly, while one also showed delayed myelination. The younger patients in the cohort did not have any neuroimaging done given the milder clinical presentation. However, it is likely that once the diagnosis of a *SIN3A*-related disorder is made, imaging of the brain should be offered in the context of neurological symptoms rather than routine work-up.

### Craniofacial dysmorphism

Our previous study presented evidence for a characteristic facial appearance associated with *SIN3A*. Some of the patients in this cohort have clear similarities. As with many of the mild and variable neurodevelopmental phenotypes, it remains to be seen whether the facial gestalt is easily identifiable in clinical practice. However, there appears to be a common, emerging facial phenotype with a tall, broad forehead, down-slanting palpebral fissures, triangular face with a pointed chin and a thin upper lip, based on the patient’s photographs (both included and unpublished but shared with the authors due to parental consent for publication of photos being declined).

Interestingly, one patient was first suspected of progeria, because of the typical shape of his neurocranium (Patient 25: J). Following genetic testing, he was diagnosed with a variant in *SIN3A*, demonstrating that this may be part of the spectrum of the syndrome. Patient 12: G also appears to have a progeric face. Sparse hair and reduced subcutaneous tissue was reported in 3/28 (10%) of patients in this study and note the progeric appearance in at least two of the patients in this cohort. However, no other ectodermal features were identified. This leads to the possibility of differential diagnoses including progeroid group of conditions; however, *SIN3A*-related disorder does not appear to present predominantly with a progeroid phenotype from the large cohort described here.

### Other clinical manifestations

14/28 (50%) patients in our study population had either epilepsy, hypotonia, or both. Two out of the three patients with epilepsy who had a brain MRI (of a total of five patients with epilepsy) had abnormalities found. None of the patients with epilepsy from this cohort reported any psychiatric disease.

Interestingly, other commonly reported symptoms in patients with intellectual disability were not reported in our patient population. Constipation for instance, was only reported in one patient, while hearing loss and refraction abnormalities were also not as prevalent as in other intellectual disability cohorts or the 15q24 microdeletion syndrome [15, 16].

Further information also needs to be collected to ascertain whether malignancy is a significant association or merely an observation with a large cohort of patients with Witteveen–Kolk Syndrome. In addition, as described above, additional features appear to be emerging from the larger cohort of patients published here and further follow-up is required to see if this is a consistent part of the phenotype.

## Conclusion

Patients with disease causing variants in *SIN3A* usually have mild global developmental delay/ID, with some even having tested IQs in the normal range with variable penetrance. There are similar facial features in around half of patients for which a targeted molecular evaluation would be feasible. However, it is likely that diagnostic evaluation and identification of *SIN3A* variants in suspected individuals will be performed using large ID panels or WES/WGS. There is evidence to suggest these patients are at risk for psychiatric- and neurological conditions and therefore, a multidisciplinary team approach should be considered in caring for these patients. Data collected so far seems to suggest additional features such as hypotonia, seizures along with the previously well described neurodevelopmental association with this disorder.

There is no apparent genotype–phenotype correlation and/ or missense variant hot spot within *SIN3A* and the missense variants appear to be distributed throughout the gene based on observation of this cohort. As expected, majority of patients in published literature and this cohort appear to have truncating variants reinforcing haploinsufficiency as likely mechanism of pathogenicity, although *SIN3A* missense variants affecting critical functional domain in SIN3A also appear to be associated with disease. Further studies of this nature are required to ascertain clinical correlation in this disorder.

### Supplementary information

Supplementary Table 4
